# Identification of Immune-Cell-Related Prognostic Biomarkers of Esophageal Squamous Cell Carcinoma Based on Tumor Microenvironment

**DOI:** 10.3389/fonc.2021.771749

**Published:** 2021-10-25

**Authors:** Yiyao Cui, Ruiqin Hou, Xiaoshuo Lv, Feng Wang, Zhaoyan Yu, Yong Cui

**Affiliations:** ^1^ Department of Thoracic Surgery, Beijing Friendship Hospital, Affiliated to the Capital University of Medical Sciences, Beijing, China; ^2^ Department of Blood Transfusion, Peking University People’s Hospital, Beijing, China; ^3^ Department of Otorhinolaryngology, Shandong Public Health Clinical Center, Jinan, China

**Keywords:** esophageal squamous cell carcinoma, LASSO regression, WGCNA, tumor microenvironment, prognostic biomarkers

## Abstract

**Background:**

Esophageal squamous cell carcinoma (ESCC) is one of the most fatal cancers in the world. The 5-year survival rate of ESCC is <30%. However, few biomarkers can accurately predict the prognosis of patients with ESCC. We aimed to identify potential survival-associated biomarkers for ESCC to improve its poor prognosis.

**Methods:**

ImmuneAI analysis was first used to access the immune cell abundance of ESCC. Then, ESTIMATE analysis was performed to explore the tumor microenvironment (TME), and differential analysis was used for the selection of immune-related differentially expressed genes (DEGs). Weighted gene coexpression network analysis (WGCNA) was used for selecting the candidate DEGs. Least absolute shrinkage and selection operator (LASSO) Cox regression was used to build the immune-cell-associated prognostic model (ICPM). Kaplan–Meier curve of survival analysis was performed to evaluate the efficacy of the ICPM.

**Results:**

Based on the ESTIMATE and ImmuneAI analysis, we obtained 24 immune cells’ abundance. Next, we identified six coexpression module that was associated with the abundance. Then, LASSO regression models were constructed by selecting the genes in the module that is most relevant to immune cells. Two test dataset was used to testify the model, and we finally, obtained a seven-genes survival model that performed an excellent prognostic efficacy.

**Conclusion:**

In the current study, we filtered seven key genes that may be potential prognostic biomarkers of ESCC, and they may be used as new factors to improve the prognosis of cancer.

## Introduction

There are more than half a million new esophageal cancer cases diagnosed each year, leading it to be one of the most universal cancers (1). Esophageal cancer has two main subtypes: esophageal squamous cell carcinoma (ESCC) and esophageal adenocarcinoma (EAC) (2). In Asia, most esophageal cancer is ESCC and had higher mortality rates than other regions (3). Unfortunately, distal metastases have occurred in a great mass of ESCC patients when diagnosed, and the 5-year survival rate is <30% (1). Hence, it is necessary to identify novel biomarkers as improving the prognosis and providing new targets for the therapy of ESCC.

In recent years, the tumor microenvironment (TME) has attracted public attention as a novel therapeutic strategy (4). TME contains numerous cells and acts a vital role in the development and invasion of cancers (5). With the development of tumor immunology, an in-depth understanding of TME is essential to improve immunotherapy (6, 7). More recently, an algorithm called ESTIMATE was able to calculate the abundance of various immune cells in a tissue based on high-throughput second-generation sequencing data (8, 9). This provides a powerful aid for the mining and analysis of the existing numerous cancer data. There were also many research focus on the TME in cancers (10–12).

**Figure 1A:**
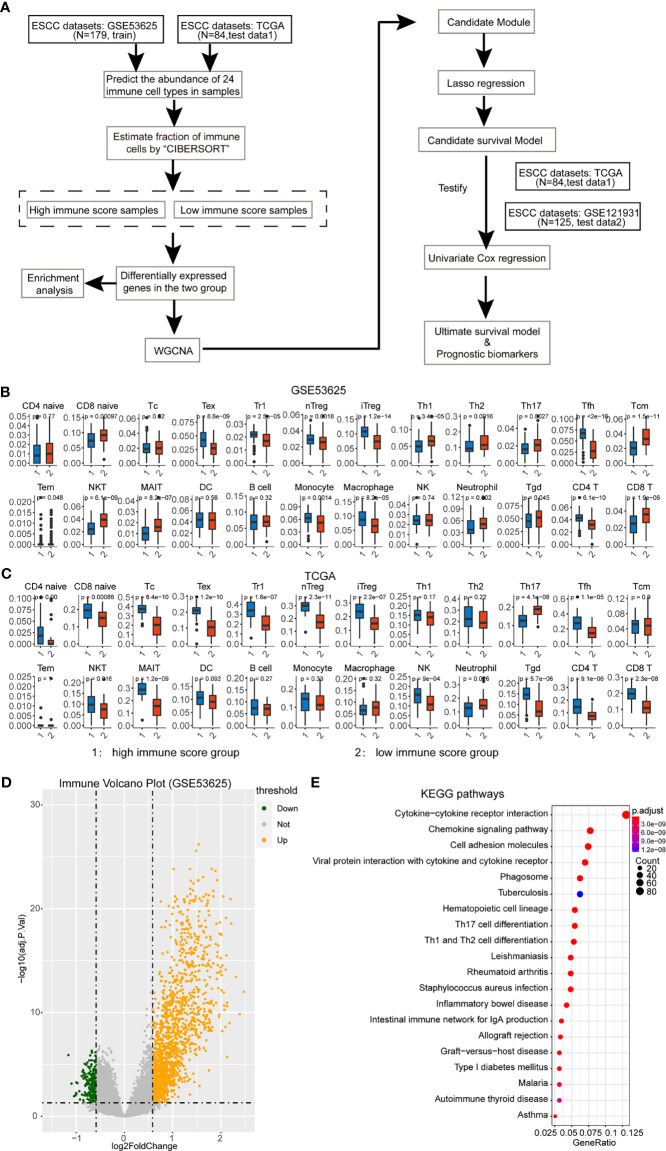
Assessment of immune cell abundance and the tumor microenvironment in ESCC. **(A)** Flow chart of this study. **(B)** Immune cell abundance in the training dataset. The p-value is displayed at the top of the bar. We only selected the significantly different cells for further analysis. **(C)** Immune cell abundance in the test dataset. **(D)** Differential analysis of immune score based on ESTIMATE analysis. The volcano plot shows the DEGs in high and low immune-score groups. **(E)** Enrichment analysis of immune-associated DEGs.

In recent years, many algorithms based on network analysis have emerged endlessly (13). A widely used algorithm, called weighted gene coexpression network analysis (WGCNA), could calculate the relationship between genes and patients’ information (14). The advantage of WGCNA is that genes with analogous patterns in expression level can be synthesized into coexpression modules and then associated with clinical characteristics so that genes related to specific traits can be screened by dimensionality reduction (15–17). The WGCNA can analyze data from large samples and establish key genes for further validation after identifying expression modules that are relevant to clinical characteristics (18). WGCNA provides a powerful aid in the search for clinically relevant molecular markers. Here, we screened multiple modules and genes closely related to the tumor microenvironment and immune cells and obtained possible biomarkers, which could improve the prognosis of ESCC (**Figure 1A**).

## Methods

### Data Sources

Three cohorts were used in the study. The training dataset was extracted from the GEO database (GSE53625). It contains 179 ESCC patients’ expression data and their clinical trait information. One of the test datasets was downloaded from the TGGA database. We obtained the RNA-seq data of 84 ESCC patients and corresponding clinical information. The gene expression level was normalized by a log2 conversion. Another test dataset containing 125 ESCC patients with clinical information was downloaded and used as another test dataset (GSE121931).

### Differential Expression Analysis

We used the ESTIMATE algorithm to get the immune scores of ESCC patients (19). Then, the patients were divided into high and low immune‐score groups based on the median of the score. Finally, we used the “limma” R package to perform the differential expression analyses between the two groups. The threshold of differentially expressed genes (DEGs) is adjusted p < 0.05 and |log2 fold change| ≥ 0.585.

We also applied the ImmuCellAI database to predict the abundance of 24 immune cell types in ESCC samples (20). The abundance of these immune cells in high and low immune‐score groups was analyzed and used as the trait of ESCC samples for further analysis.

### Weighted Gene Coexpression Network Analysis

We used the “WGCNA” R package to construct the coexpression network of immune-related DEGs based on the automatic network construction function. Then, gene modules with similar expression pattern hierarchical were detected. Finally, patients’ characteristics of immune cell infiltration were associated with these modules, and the key genes from the candidate module were explored for further analysis.

### Enrichment Analysis

We used the “clusterProfiler” R package for functional enrichment analysis of genes of interest. The background functions including the Kyoto Encyclopedia of Genes and Genomes (KEGG) pathways and GO terms. In the analysis, only functions with a p < 0.05 will be selected.

### Survival Analysis

The R package glmnet was applied to build an immune cell-associated prognostic model (ICPM) of ESCC patients by LASSO analysis. Subsequently, we used the survival R package to depict the survival curve to estimate the efficiency of the ICPM and the genes in the model. We analyzed the overall survival between different clinical subgroups of ESCC patients based on the risk score, too. The subgroups included age (≤59 or >59 years), gender, alcohol, tobacco, and tumor–node–metastasis (TNM) stage. The pROC package was used to assess the prognostic efficiency of the ICPM. Univariate cox regression analysis was performed to filter the prognosis-associated genes in the ICPM, and a Cox regression model was constructed based on its result.

### Statistical Analysis

All statistical analyses were calculated through R software (version 4.0.3). t-test was used to compare the differences between the selected two groups. Adjusted p < 0.05 was regarded as significant.

## Results

### Evaluation of Immune Cell Abundance in ESCC Patients and Identification of Immune-Associated DEGs

In the training dataset, we split the 179 ESCC patients into high- and low-immune score groups based on the ESTIMATE analysis. Next, we explored the immune cell abundance of the cohort. It showed that most of the 24 immune cells were different in these groups except CD4-naive, cytotoxic T cell, NKT, DC, and B cell (**Figure 1B**). Then, the test data contains 84 ESCC patients who had performed the same analysis. These immune cells had a similar abundance in the test dataset (**Figure 1C**).

Subsequently, we identified DEGs between these groups and found 1,489 differentially upregulated genes and 213 downregulated genes in the training dataset (**Figure 1D**). The enrichment analysis also showed that these DEGs were involved in the Th17 cell differentiation, Th1 and Th2 cell differentiation, cell adhesion, and so on (**Figure 1E**). It hints that the DEGs participate in the immune-associated functions in ESCC patients.

### Identification of Immune-Cells-Associated Modules

**Figure 2:**
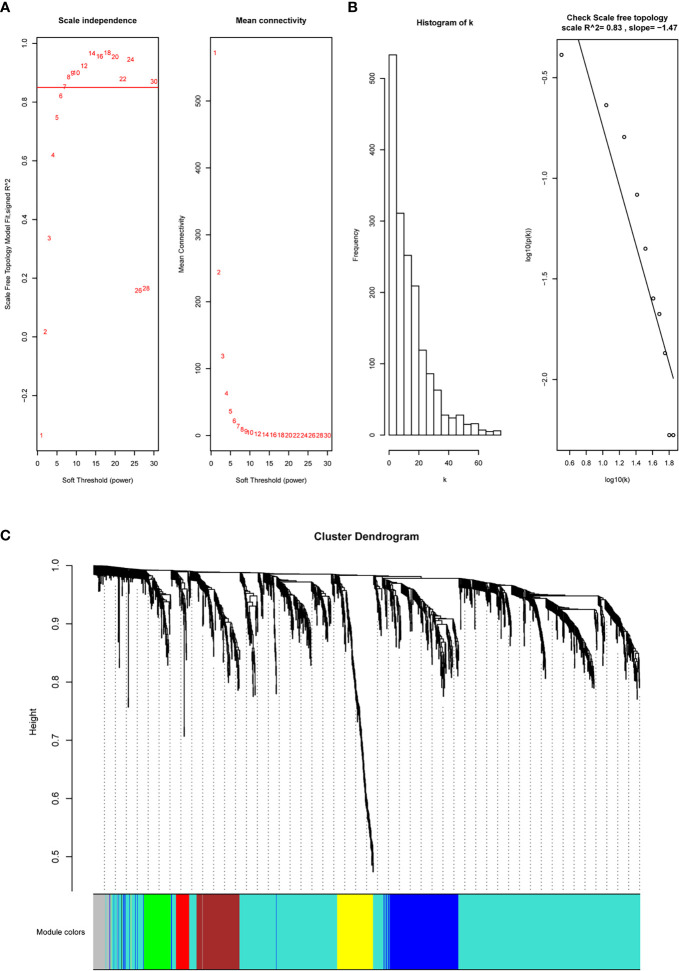
WGCNA to construct the coexpression modules based on immune-related DEGs. **(A, B)** The soft power is set at 7 based on the R^2^ of the scale-free topology model, which reached 0.82. **(C)** Six modules were clustered based on under the parameter.

**Figure 3:**
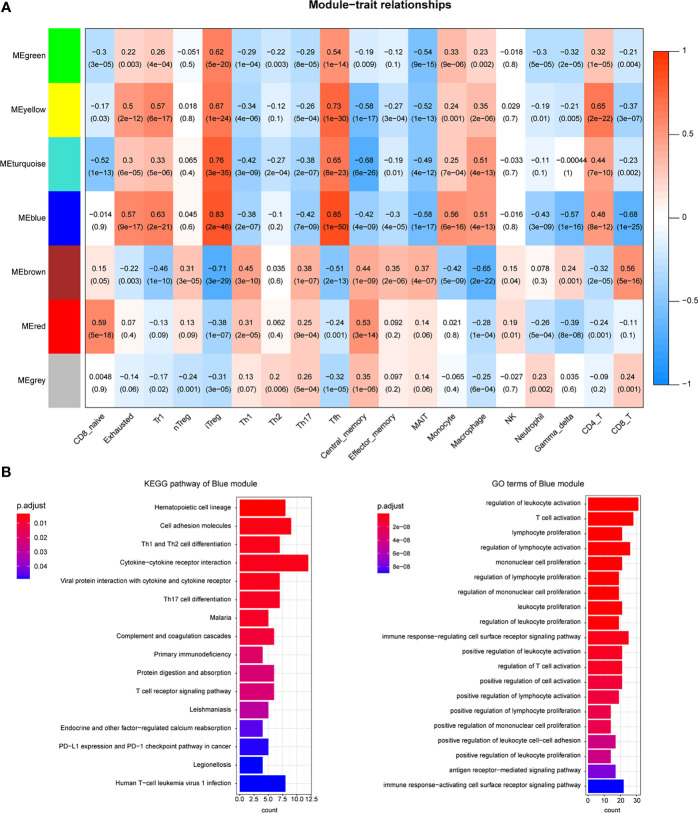
Coexpression modules were related to immune cells. **(A)** Heatmap shows the relationship between coexpression modules and immune cell abundance. **(B)** Enrichment analysis of candidate DEGs in the blue module.

The immune-related DEGs were then utilized to cluster the coexpression modules. We used the pickSoftThreshold function to calculate the cutoff of soft power. Soft power is a key parameter to cluster the modules. In the study, the power threshold was calculated at 7 (**Figure 2A**). When this power is used, the R^2^ of the scale-free topology model under the soft threshold is 0.82. It hints that the network we constructed conformed to scale-free characteristics (**Figure 2B**). Next, we constructed the network with default module size and built six coexpression modules (green, yellow, turquoise, blue, brown, and red) (**Figure 2C**). Subsequently, we calculated the correlation between these six modules and immune cell abundance to filter the most important modules and crucial genes. It showed that the blue module was significantly associated with most immune cells, especially iTreg and Tfh cells (**Figure 3A**). The blue module contained 240 genes, and the enrichment analysis showed that they are related to the T-cell receptor signaling pathway, cell adhesion, antigen-receptor-mediated signaling pathway, etc. (**Figure 3B**). The enrichment results hinted that these genes are immune-associated in the tumor microenvironment of ESCC.

### Construct the Immune-Cell-Associated Prognostic Model

**Figure 4:**
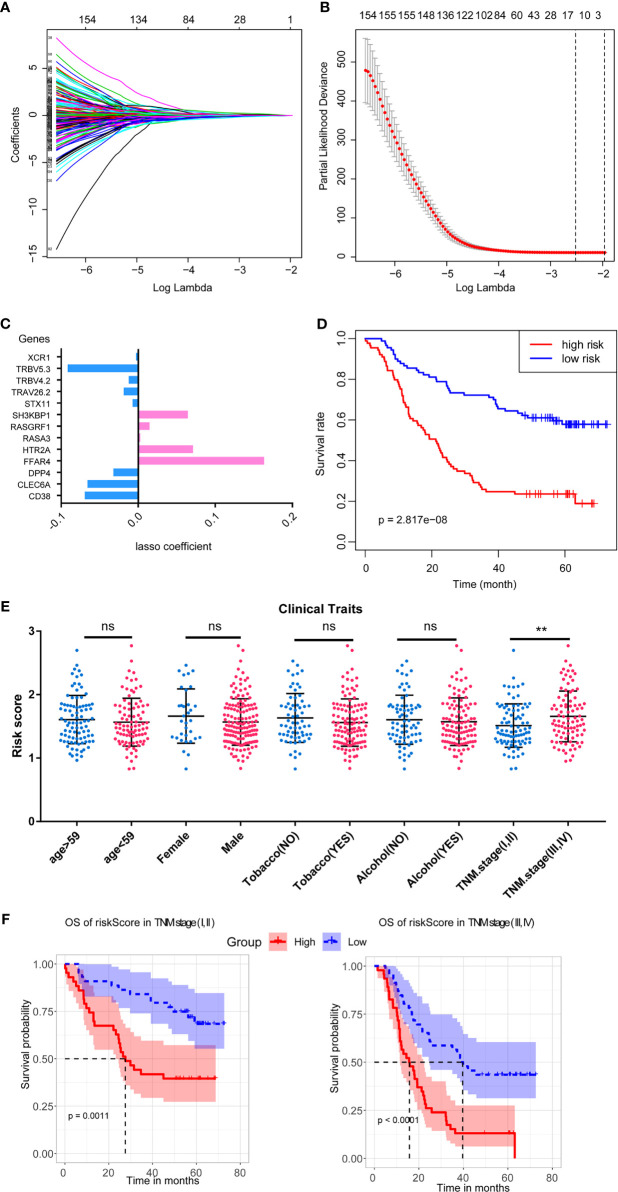
LASSO regression to construct the ICPM and its stratified survival analysis. **(A, B)** The best criteria to build the model based on LASSO regression. **(C)** The coefficients of the candidate genes in the survival model. **(D)** The ICPM has an excellent prognostic efficacy. **(E)** The ICPM can separate patients with different TNM stages into different subgroups based on risk score. **(F)** The ICPM could predict the prognosis in patients with different TNM stages. ns, not significant, **p < 0.01.

To select the potential prognostic genes in the blue module, we used LASSO analysis on the 240 genes to generate an ICPM. This is the best model, which contains 13 genes based on LASSO analysis (**Figures 4A, B**). **Figure 4C** shows the coefficient of the 13 genes in the LASSO model. Subsequently, survival analysis revealed that high-risk-score patients had less overall survival time (**Figure 4D**). We then analyzed the relationship between the patients’ clinical traits and the model to evaluate the ICPM’s efficacy. We found that this model could distinguish the TNM stage (**Figure 4E**). Thus, a stratified survival analysis was used to access the prognostic efficacy in different TNM stages. Interestingly, high-risk-score patients also had a bad prognosis in TNM stages (**Figure 4F**). The result substantiated that the model could be used as a predictor and helps to improve the prognosis of ESCC.

### Survival Analysis of the 13 Genes in ICPM

**Figure 5:**
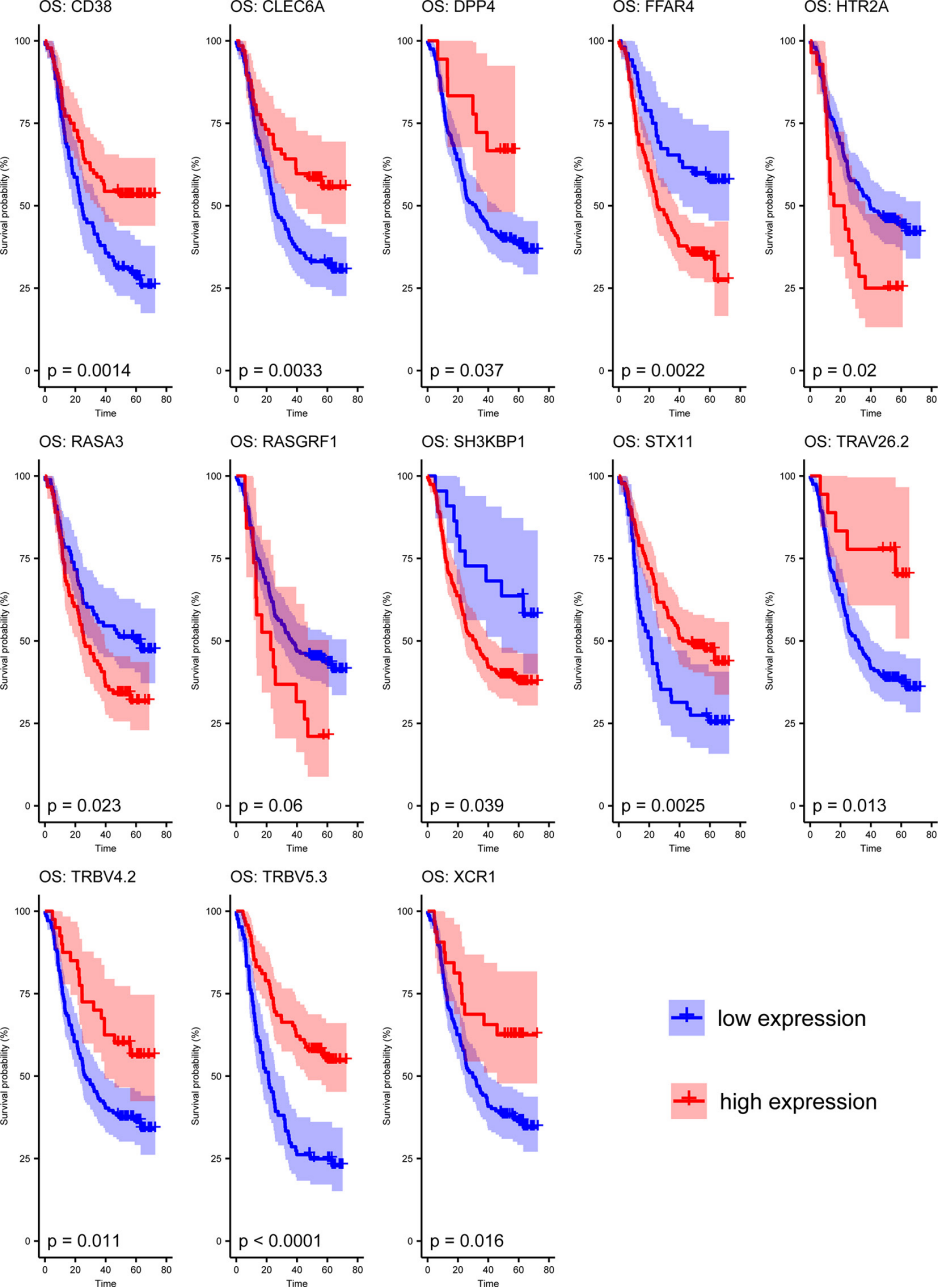
Survival analysis of the 13 candidate genes in ICPM.

The 13 crucial genes in ICPM were then tested by survival analysis. It indicated that most of these genes are prognosis associated in the training dataset except RASGRF1 (**Figure 5**). To be specific, 4 out of the crucial 13 genes (FFAR4, HTR2A, RASA3, and SH3KBP1) were risk factors of ESCC patients, while the other 8 genes (CD38, CLEC6A, DPP4, STX11, TRAV26-2, TRBV4-2, TRBV5-3, and XCR1) are protective factors.

Then, two test datasets were used to test the 13 genes’ survival ability. In the Cancer Genome Atlas (TCGA) test dataset, eight genes were detected, and six of them were survival associated (DPP4, FFAR4, RASA3, RASGRF1, XCR1, and STX11) (**Supplementary Figure 1**). In another test dataset with 125 ESCC patients, 10 genes were detected, and 6 of them were survival associated (CLEC6A, DPP4, HTR2A, RASA3, SH3KBP1, and XCR1) (**Supplementary Figure 2**).

### Construct the Ultimate Survival Model Based on the ICPM

**Figure 6:**
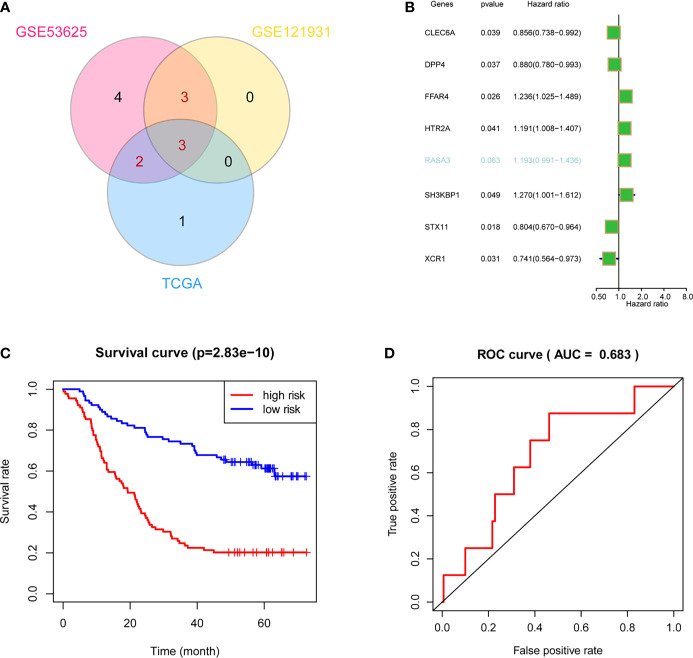
Validation and optimization of ICPM through multidatasets. **(A)** Stable-survival-associated candidate genes in three cohorts. **(B)** Cox regression analysis showed that six candidate genes were independent prognostic factors. **(C)** The optimized survival model has a good effect on ESCC patients’ prognosis. **(D)** AUC indicates the well-predicted efficacy of the optimized model.

After the filtration by two test datasets, we obtained eight stable prognostic genes that were survival associated in at least two datasets (**Figure 6A**). Next, we used univariate Cox regression analysis to select independent prognostic genes. Seven out of the eight crucial genes were independent prognostic factors of overall survival (OS) in ESCC patients (**Figure 6B**). Thus, we used the seven genes to construct a survival model. It showed that the model performed an excellent efficacy in prognosis (**Figure 6C**), and the receiver operating characteristic (ROC) curves also proved the efficacy of the model (**Figure 6D**).

## Discussion

ESCC is one of the most malignant cancers in the world. However, few biomarkers can accurately predict the prognosis of patients with ESCC (21). In addition, some studies have already demonstrated the effect of immune cells on cancers, and the immune cells in ESCC can be applied to access its therapeutic and prognostic effects (10, 22). In the current study, by performing an integrated analysis of immune microenvironment and gene expression pattern, we investigated potential prognostic biomarkers in ESCC base on the following steps: (1) predict the immune cell abundance in ESCC, (2) assess the tumor microenvironment of ESCC and identify differentially expressed genes, (3) enrich DEGs through Gene Ontology and KEGG pathway analysis, (4) construct the coexpression network of immune-related DEGs through WGCNA, (5) associate immune cell infiltration and coexpression modules, (6) construct the survival model, and (7) validate the model and improve its efficacy.

The tumor microenvironment is important for the study of immune-related target molecules and prognostic markers (23, 24). There have been many studies using the tumor microenvironment to search for prognostic markers (25–27). Furthermore, the integration of multiple bioinformatics analyses has been widely used with ESCC (28). Thus, the integration of immune cells and ESCC is feasible in the discovery of prognostic biomarkers. In recent years, immunotherapy has been recognized as a treatment strategy that performs well in many types of cancer (29). It works against cancer cells by inhibiting immune checkpoints. The development of monoclonal antibodies that inhibit programmed death 1 (PD-1) or programmed death-ligand 1 (PD-L1) has also been shown to produce convincing clinical efficacy in a variety of malignancies, including ESCC (30). However, the efficacy of these drugs is limited. Therefore, there is an urgent need to identify new biomarkers associated with immune cells to select patients sensitive to these drugs and thus improve the prognosis of ESCC.

Here, we obtained seven crucial genes that may act as the prognostic factor of ESCC. CLEC6A is a gene of human innate immunity. It can directly mediate intracellular signaling, recognize a variety of endogenous and exogenous ligands, and drive both innate and adaptive immunity (31). DPP4 is an inherent type II transmembrane glycoprotein and serine peptidase involved in glucose and insulin metabolism and immune regulation (32). DPP4 has been found to participate in thyroid papillary carcinoma cell proliferation by inhibiting the mitogen-activated protein kinase (MAPK) pathway (33). FFAR4 encodes G-protein-coupled receptor (GPR) and is involved in anti-inflammatory responses (34). FFAR4 can promote cell proliferation and migration and has been identified as a potential prognostic biomarker for laryngeal cancer (35). HTR2A encodes one of the serotonin receptors and could activate the PI3K-Akt-MTOR signal (36). SH3KBP1 is involved in several cellular processes, such as apoptosis, cytoskeletal rearrangement, and cell adhesion (37). It has been reported to promote tumor proliferation and invasion in ESCC (38). STX11 is involved in intracellular vesicle transport. It has been found to play a tumor-suppressive role in peripheral blood T-cell lymphoma (39). XCR1 signals by increasing intracellular calcium levels. It has been found to promote the migration of non-small cell lung cancer (40). At present, there are no key targets for the prognosis of ESCC. Although these genes are not specifically expressed in ESCC, they have been able to show the potential ability to predict the prognosis of cancer through the integration of the survival model, which is helpful to determine its prognostic targets. Moreover, subsequent studies can further determine the expression levels of these key genes at low throughput levels and further study their important mechanisms in esophageal cancer. In short, our study provides a solid foundation for subsequent prognostic and mechanism research.

All in all, our findings provide several potential prognostic biomarkers of ESCC and may improve the treatment of this type of cancer.

## Data Availability Statement

The original contributions presented in the study are included in the article/**Supplementary Material**. Further inquiries can be directed to the corresponding authors.

## Author Contributions

YiC, ZY, and YoC designed the whole study. YiC and RH performed most of the analysis. XL and FW collected the high throughput data. ZY search the analysis tools. YiC wrote the manuscript. All authors contributed to the article and approved the submitted version.

## Funding

This work was supported by a grant from Wu Jieping Medical Foundation (320.6750.2020-17-4) and project (RDE2019-18) supported by Peking University People’s Hospital Scientific Research Development Funds.

## Conflict of Interest

The authors declare that the research was conducted in the absence of any commercial or financial relationships that could be construed as a potential conflict of interest.

## Publisher’s Note

All claims expressed in this article are solely those of the authors and do not necessarily represent those of their affiliated organizations, or those of the publisher, the editors and the reviewers. Any product that may be evaluated in this article, or claim that may be made by its manufacturer, is not guaranteed or endorsed by the publisher.

## Supplementary Material

Supplementary Figure 1 Survival analysis of the thirteen candidate genes of ICPM in TCGA dataset.

Supplementary Figure 2 Survival analysis of the thirteen candidate genes of ICPM in another test dataset.
