# Supplementary figures and images for: Identification of Immune-Cell-Related Prognostic Biomarkers of Esophageal Squamous Cell Carcinoma Based on Tumor Microenvironment
Source: Front Oncol. 2021 Oct 25;11:771749. doi: 10.3389/fonc.2021.771749 (PMC8573319; doi:10.3389/fonc.2021.771749)

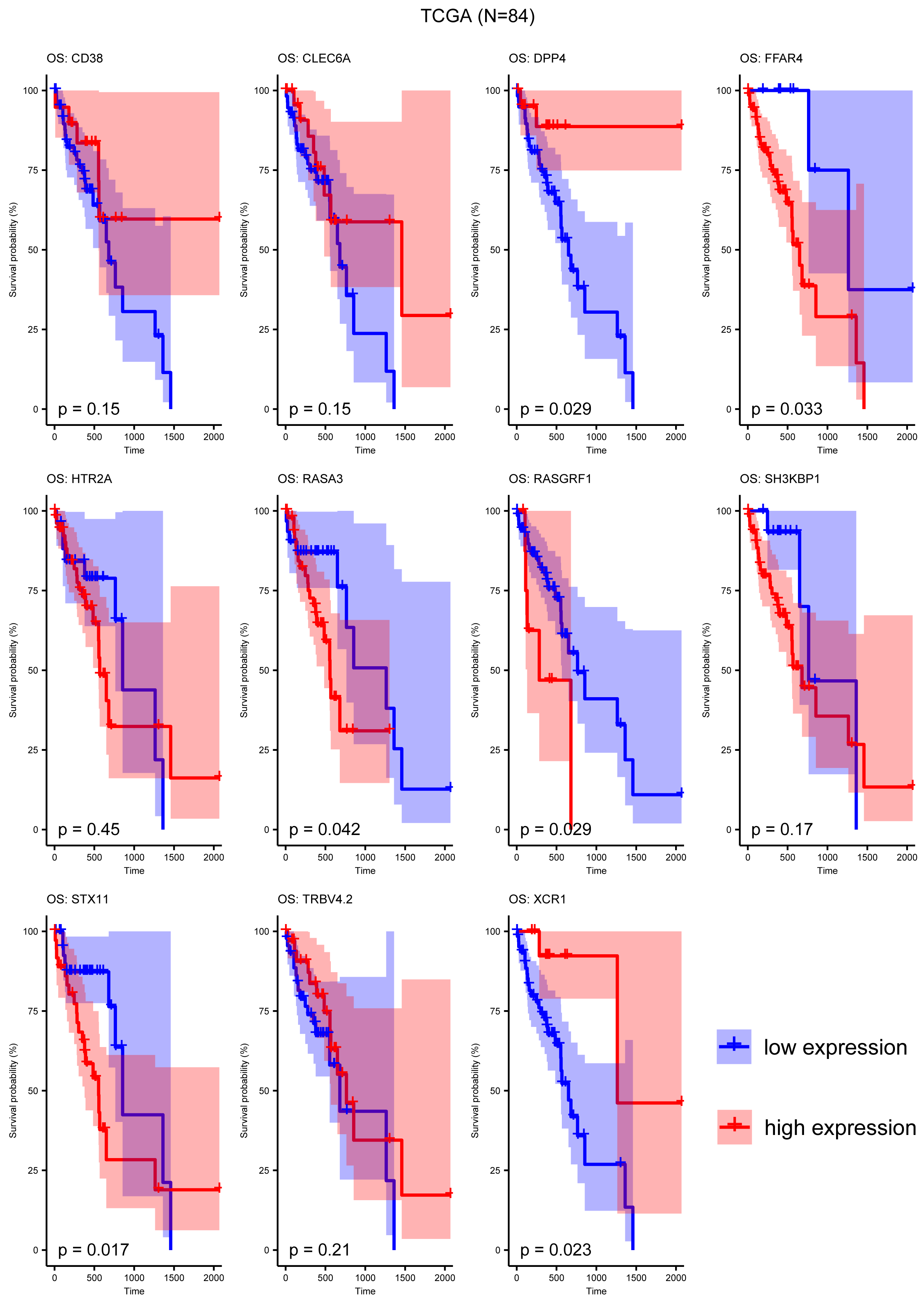

Supplement: Supplementary Figure 1 — Survival analysis of the thirteen candidate genes of ICPM in TCGA dataset. [file Image_1.tif]

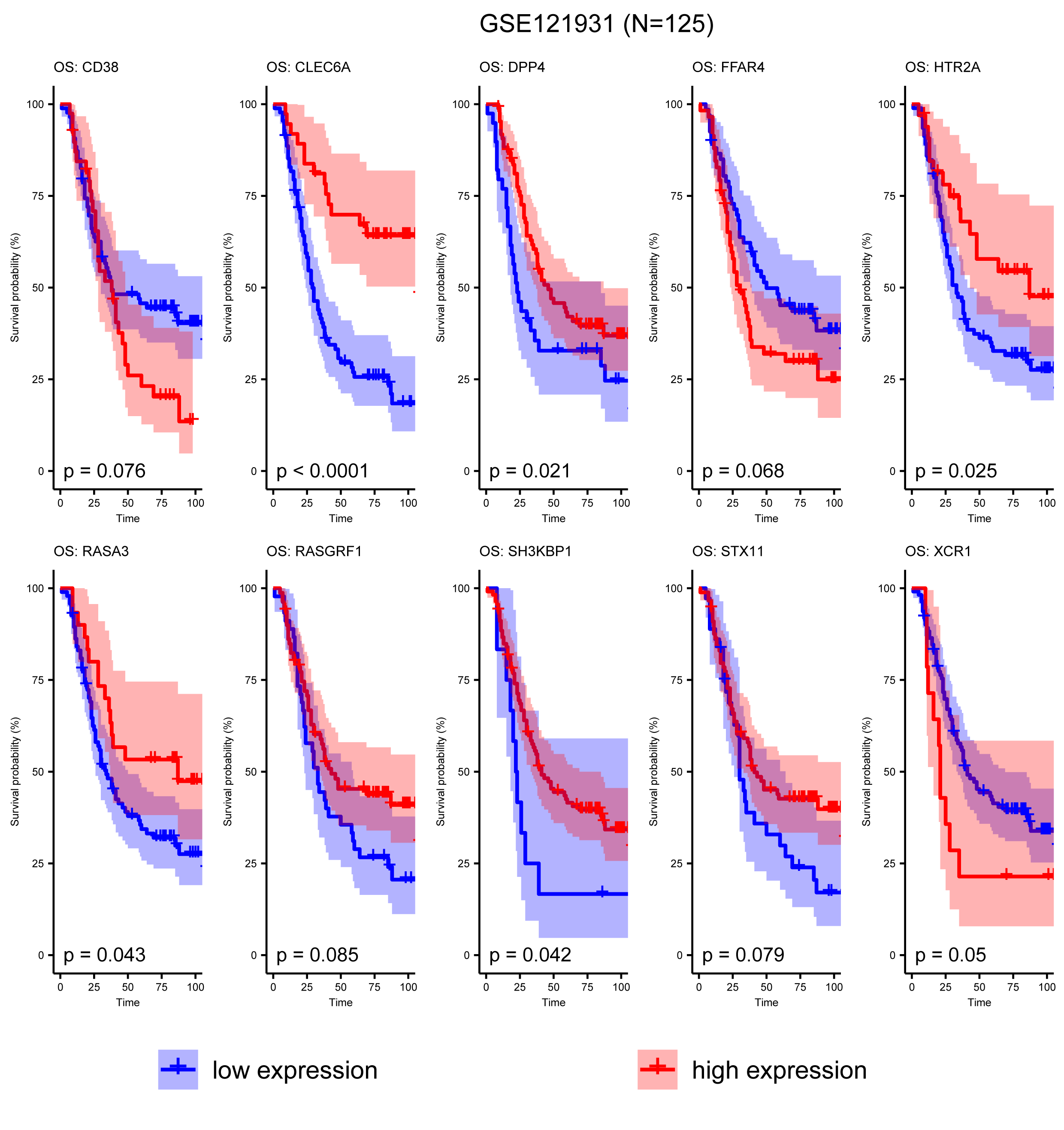

Supplement: Supplementary Figure 2 — Survival analysis of the thirteen candidate genes of ICPM in another test dataset. [file Image_2.tif]
